# Isolation and Purification of Novel Antioxidant Peptides from Mussel (*Mytilus edulis*) Prepared by Marine *Bacillus velezensis* Z-1 Protease

**DOI:** 10.3390/md23080294

**Published:** 2025-07-23

**Authors:** Jing Lu, Pujing Shi, Yutian Cao, Bingxin Shi, Huilin Shen, Shuai Zhao, Yuchen Gao, Huibing Chi, Lei Wang, Yawei Shi

**Affiliations:** 1 College of Life Sciences, Shanxi University, Taiyuan 030006, China; jinglu@sxu.edu.cn (J.L.); shipujing@sxu.edu.cn (P.S.); 13303564766@163.com (Y.C.); 19888598200@163.com (B.S.); shl_1110@163.com (H.S.); zhaoshuai@sxu.edu.cn (S.Z.); gaoyuchen@sxu.edu.cn (Y.G.); 2 College of Food Science and Technology, Nanjing Agricultural University, Nanjing 210095, China; t2023091@njau.edu.cn; 3 Key Laboratory of Chemical Biology and Molecular Engineering, Ministry of Education, Institute of Biotechnology, Shanxi University, Taiyuan 030006, China

**Keywords:** marine bioactive peptides, mussel hydrolysate, *Bacillus velezensis*, antioxidant activity, molecular docking

## Abstract

Mussels are nutrient-rich but perishable, resulting in substantial resource loss. A protease-producing strain (*Bacillus velezensis* Z-1, *Mytilus edulis*) isolated from marine sludge was used to hydrolyze mussels, producing Y-1, a hydrolysate with antioxidant activity. In this study, ultrafiltration, gel chromatography, and LC-MS/MS were employed to isolate and identify bioactive peptides from the hydrolysate. The results revealed that the hydrolysate exhibited antioxidant activity, pancreatic cholesterol esterase inhibitory activity, pancreatic lipase inhibitory activity, and α-glucosidase inhibitory activity. Molecular docking using AutoDock Tools 1.5.6 was performed to analyze the interactions of peptides with CD38 and Keap1, leading to the identification of five potentially bioactive peptides: VPPFY, IMLFP, LPFLF, FLPF, and FPRIM. These peptides formed hydrogen bonds and hydrophobic interactions with CD38 and Keap1, demonstrating strong DPPH radical scavenging and superoxide anion radical scavenging capacities. This study highlights the multifunctional bioactive potential of these peptides, offering insights into their therapeutic applications. The findings provide a novel approach for the effective utilization of mussel resources and highlight their potential application value in the development of functional foods.

## 1. Introduction

Bioactive peptides are defined as specific oligomeric amino acid fragments derived from natural resources, typically composed of 3 to 20 amino acid residues. These molecules exhibit a wide spectrum of physiological and pathological regulatory functions [1]. As efficient modulators of multiple biological processes, polypeptides have emerged as a promising class of therapeutic agents, with over 80 peptide-based drugs currently available on the market [2]. Notably, bioactive peptides are generally regarded as safer than conventional small-molecule drugs and demonstrate superior human absorbability. The ocean, hosting a vast and diverse array of organisms, serves as an exceptional reservoir for the discovery and preparation of bioactive peptides. Accumulating evidence has confirmed that marine-derived bioactive peptides can activate the Keap1–Nrf2 signaling pathway [3], a key regulator of cellular antioxidant responses. For instance, Tongxin Zhi et al. revealed that the antioxidant capacity of scallop-derived peptides is closely associated with the content and positional distribution of aromatic, basic, and acidic amino acids [4]. Concurrently, Zixu Wang et al. reported that scallop protein hydrolysates exhibit potent antioxidant activity, along with excellent foaming and emulsifying properties [5]. These peptides not only effectively inhibit lipid oxidation but also provide robust protection against H_2_O_2_-induced cytotoxicity in vitro.

During normal human physiological processes, free radical generation and elimination stay in dynamic balance. When this balance is disrupted, oxidative stress occurs, leading to diseases like cancer, atherosclerosis, and various chronic ailments. Antioxidant bioactive peptides can reduce free radical-induced body damage and show stronger antioxidant activity than their parent proteins, holding great potential for food and pharmaceutical applications [6,7,8]. For example, the WPP peptide from clam muscle hydrolysate has EC50 values of 1.39, 0.41, 0.54, and 2.75 mg/mL for scavenging DPPH, hydroxyl, superoxide anion, and ABTS radicals, while the LFKKNLLTL peptide from Ruditapes philippinarum muscle hydrolysate lowers ROS levels and increases glutathione in HepG2 cells [9]. Antioxidant peptides from rainbow trout viscera hydrolysis exhibit 80.41 ± 0.21% DPPH and 90.32 ± 0.35% ABTS radical scavenging rates [10], and oyster-derived polypeptides DS9 and HF6 protect MC3T3-E1 cells from H_2_O_2_ damage, with antioxidant and anti-osteoporotic effects [11]. Besides antioxidant functions, bioactive peptides also exert significant effects in blood glucose regulation, anti-inflammation and anticancer activities [12,13,14,15,16].

Mussels, belonging to the class Bivalvia within the phylum Mollusca, are nutritionally dense, containing high levels of proteins, omega-3 polyunsaturated fatty acids (PUFAs), iodine, and carbohydrates. These bivalves exhibit multiple biological activities, including antibacterial, antioxidant, antihypertensive, anticoagulant, and anti-inflammatory effects, and have been shown to protect against oxidative damage in HepG2 cells and acute alcoholic liver injury in mice [17]. Bioactive peptides extracted from mussels hold promise for various applications: they aid in disease prevention and treatment, promote human health, facilitate efficient utilization of marine resources, reduce environmental pollution, and contribute to coordinated economic and environmental development [18]. A recent study isolated the protease-producing strain *Bacillus velezensis* Z-1 (deposit number CGMCC No. 25059) from marine sludge, which hydrolyzes mussels to yield the antioxidant hydrolyzate Y-1 [19]. Using a combination of gel chromatography, ultrafiltration, and LC-MS/MS, bioactive peptides in the hydrolyzate were isolated and identified. Molecular docking via Autodock software analyzed interactions between these peptides and the targets CD38 and Keap1, screening for peptides with potential antioxidant activity, whose capacities were experimentally verified. This work aims to identify functional bioactive substances from mussel resources, providing a novel approach for their effective utilization and highlighting their potential in developing functional foods and pharmaceuticals.

## 2. Results

### 2.1. Separation, Purification, and Activity Determination of Y-1

**Figure 1:**
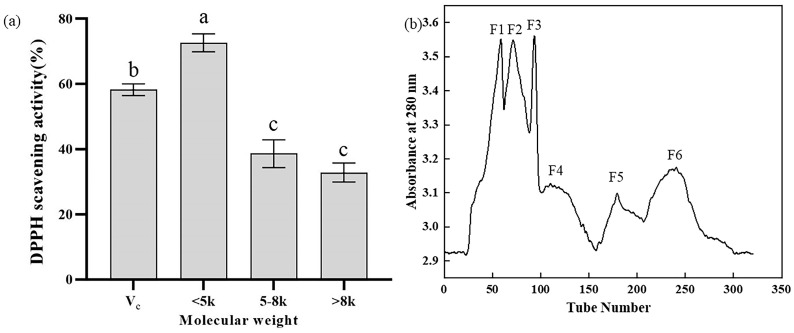
(**a**) DPPH scavenging activities of different components of hydrolysate Y-1 (%), (**b**) elution curve for molecular weight < 5 kDa on Sephadex G-25 column. Different lowercases above the error bar suggest obvious differences in antioxidant activity of separated components (*p* < 0.05).

Ultrafiltration was employed to fractionate the Y-1 hydrolysate into three molecular weight fractions: >8 kDa, 5−8 kDa, and <5 kDa. As depicted in Figure 1, bioactive peptides of varying molecular weights exhibited distinct effects on DPPH radical scavenging activity (Figure 1a). Notably, the <5 kDa fraction of the mussel hydrolysate demonstrated a DPPH scavenging rate exceeding 70%, outperforming both higher molecular weight fractions and 10 mg/mLV_C_, which underscores its optimal antioxidant activity. To further isolate the most active components, a Sephadex G-25 chromatographic column (100 cm × 1.6 cm) was used to fractionate the <5 kDa peptide fraction. This separation yielded six distinct absorption peaks (Figure 1b), and the eluates corresponding to each peak were collected and freeze-dried for subsequent analysis.

### 2.2. Determination of In Vitro Activities

**Figure 2:**
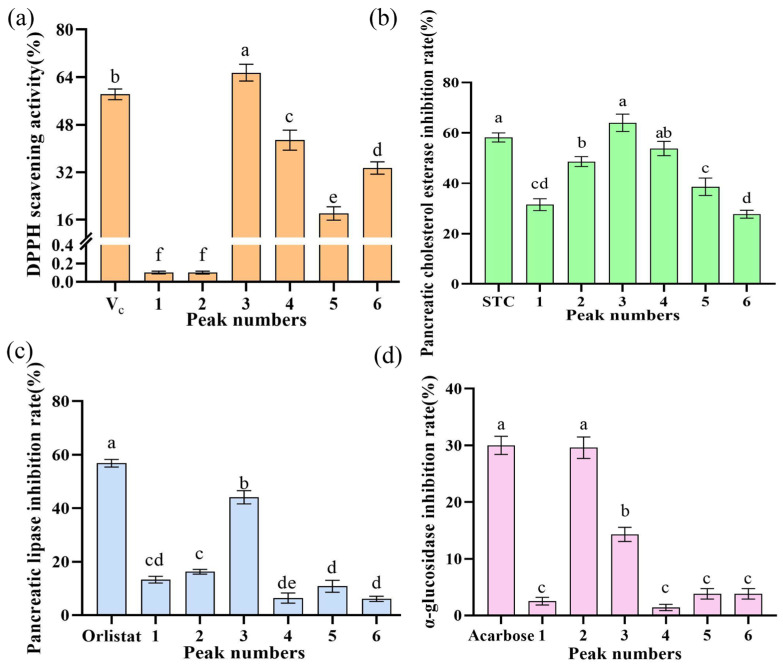
(**a**) DPPH radical scavenging rate; (**b**) pancreatic cholesterolase inhibitory activity; (**c**) pancreatic lipase inhibitory activity; (**d**) α-glucosidase inhibitory activity. Different lowercases above the error bar suggest obvious differences in antioxidant activity of separated components (*p* < 0.05).

The DPPH radical scavenging activity, pancreatic cholesterol esterase inhibitory activity, pancreatic lipase inhibitory activity, and α-glucosidase inhibitory activity of different absorption peaks are presented in Figure 2. Notably, Peak 1 and Peak 2 showed negligible DPPH scavenging capacity, whereas Peak 3 exhibited the most pronounced DPPH scavenging effect (Figure 2a), outperforming 10 mg/mL V_C_. This peak also demonstrated significant pancreatic cholesterol esterase and lipase inhibitory activities (Figure 2b,c), with cholesterol esterase inhibition (64.0%) exceeding that of 4 mg/mL sodium taurocholate (58.2%), though lipase inhibition (44.1%) was weaker than 2 mg/mL orlistat (56.8%). In contrast, Peak 2 displayed the highest α-glucosidase inhibitory activity (29.6%, Figure 2d), comparable to 1 mg/mL acarbose. Following comprehensive activity evaluation, Peak 3 was identified as the most bioactive fraction and subjected to LC-MS/MS analysis for peptide sequence identification.

### 2.3. Identification of Peptide Sequences

Peptide identification for Peak 3 was performed by Wuhan Dangang Biotechnology Co., Ltd., with the corresponding protein chromatogram shown in Figure S1. A total of 384 peptide sequences were obtained, which were ranked by descending order using the comprehensive scoring system of the PeptideRanker website (detailed in Table S1). Most sequences consisted of 5–6 amino acids with molecular weights all below 1 kDa, indicating that this fraction primarily contains small peptide segments. As reported [20], small peptides show higher reactivity with free radicals for electron transfer compared to larger fragments, endowing low-molecular-weight peptides with enhanced antioxidant activity and diverse bioactive potentials. Based on these features, a computer-aided simulation approach was employed for preliminary screening of the polypeptides.

### 2.4. Screening and Characterization of Bioactive Peptides

Peptides were screened using two computational platforms: those with a PeptideRanker score > 0.5 (http://distilldeep.ucd.ie/PeptideRanker/, accessed on 17 March 2024) or a PepCalc comprehensive score > 85 were considered potential antioxidant candidates. The BIOPEP-UMW database (https://biochemia.uwm.edu.pl/en/biopep-uwm-2/, accessed on 17 March 2024) was used to verify novelty: peptides were deemed previously reported if they showed >30% sequence identity to known antioxidant peptides [21].The functional attributes and antioxidant activity of peptides are intricately correlated with the amino acid composition. Amino acid residues such as Ile, Leu, Val, Tyr, Phe, His, and Asp function as efficacious proton donors and antioxidant amino acid residues [22]. The presence of a significant quantity of amino acids, including Phe, Ile, Pro, Val, Leu, and Met, within the polypeptide fraction augments the probability of its antioxidant potential. Yufeng Duan obtained a product through the hydrolysis of beef liver using alkaline protease and papain, wherein the contents of Val, Ile, and Leu were relatively elevated and exhibited a statistically significant difference (*p* < 0.05) compared to those obtained via hydrolysis with other enzymes. Met, being a sulfur-containing amino acid, is capable of furnishing sulfhydryl groups and may thus possess an effective free radical scavenging function [23]. Ile, Pro, Val, Leu, and Met are essential amino acids, while Phe is both an essential and an aromatic amino acid. These amino acids can donate protons to stabilize electron-deficient free radicals and either maintain their stability or enhance their interaction with free radicals, which may potentially augment their biological characteristics [24]. Based on the molecular weight, the proportion of hydrophobic amino acids, the hydrophobicity, and the binding energy of molecular docking with CD38, these 384 polypeptides were screened, and a total of five antioxidant peptides with relatively prominent potential activities were procured. Table 1 presents partial details of the screening of these five polypeptides. It is noteworthy that a lower binding energy indicates a more favorable binding effect with the CD38.

### 2.5. Molecular Docking

#### 2.5.1. The Docking of Polypeptides with CD38

CD38 is capable of degrading NAD^+^. NAD^+^ functions as a pivotal cofactor in SIRT1-7-mediated signal transduction, engaging in metabolic pathways (such as hydrogen atom transfer, partial oxidation reactions within the tricarboxylic acid cycle, and oxidation of fatty acids and amino acids in mitochondria) and cellular redox reactions. It can also specifically bind to the active centers of other enzymes, thereby regulating biological processes such as cell apoptosis, senescence, and DNA repair. It can also specifically bind to the active sites of other enzymes, thereby regulating processes such as cell apoptosis, aging and DNA repair. CD38 is a protein that degrades NAD^+^ in the body. Some antioxidant peptides tightly bind to CD38 through hydrogen bonds and hydrophobic interactions, occupying its binding site with NAD^+^, thereby preventing the binding of CD38 to NAD^+^ and inhibiting its activity. This prevents the consumption and loss of NAD^+^ in the body and ultimately prevents oxidation and aging [25].

**Figure 3:**
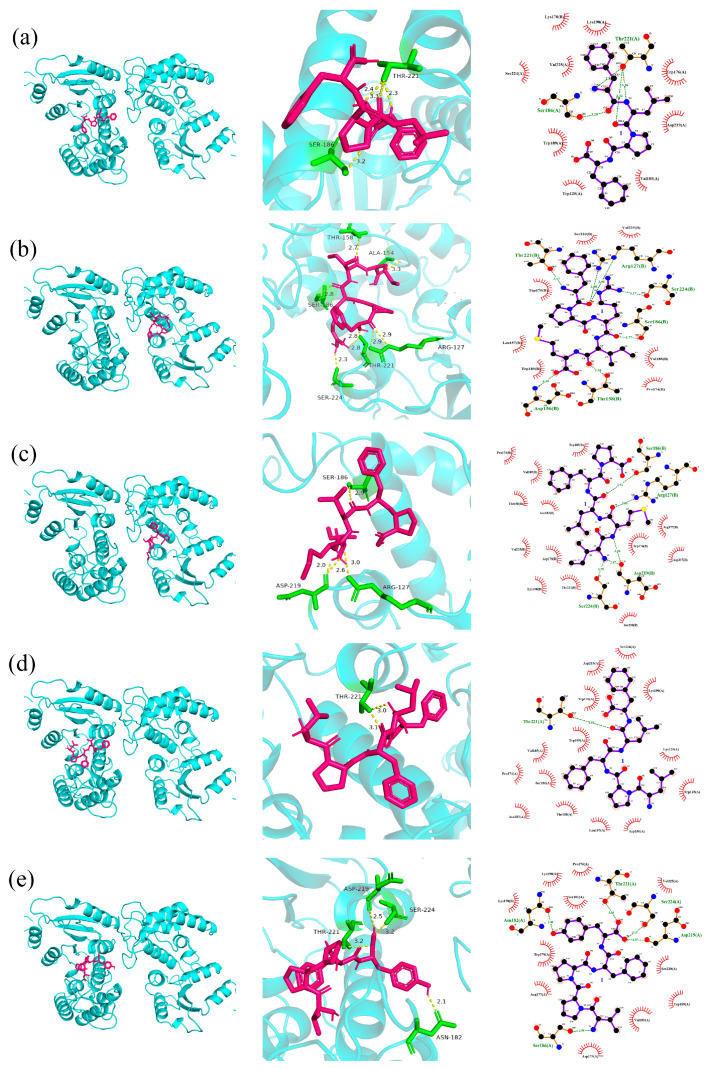
3D and 2D docking diagrams of FLPF (**a**), FPRIM (**b**), IMLFP (**c**), LPFLF (**d**), and VPPFY (**e**) with the active site of CD38. The purple part in the figure represents the ligand, the green part represents the residues of the receptor, and the yellow dotted lines represent hydrogen bonds.

FLPF establishes three hydrogen bonds, with bond lengths of 2.3 Å, 2.4 Å, and 3.3 Å, respectively, with the Thr221 residue of CD38 and a single hydrogen bond of 3.2 Å with the Ser186 residue. Concurrently, it forms nine hydrophobic interactions with the residues Lys178, Lys190, Ser224, Val225, Trp176, Trp189, Asp219, Trp125, and Val185, thereby occupying the crucial binding site of CD38 for NAD^+^ and effectively precluding their association (Figure 3a).

FPRIM forms one hydrogen bond of 2.3 Å with the Ser224 residue of CD38, two hydrogen bonds of 2.8 Å with the Thr221 residue, two hydrogen bonds of 2.9 Å with the Arg127 residue, and one hydrogen bond each with the Ser186, Asp156, and Thr158 residues, with corresponding bond lengths of 2.8 Å, 3.3 Å, and 2.7 Å. Additionally, it forms seven hydrophobic interactions with the residues Val225, Ser220, Trp176, Leu157, Val185, Trp189, and Pro174, occupying the key binding site of CD38 with NAD^+^ and impeding their binding (Figure 3b).

IMLFP forms one hydrogen bond of 2.9 Å with the Ser186 residue of CD38, one hydrogen bond of 3.0 Å with the Arg127 residue, and two hydrogen bonds of 2.0 Å and 2.6 Å with the Asp219 residue. Moreover, it forms thirteen hydrophobic interactions with the residues Pro174, Thr158, Thr221, Val185, Val225, Asn183, Asp217, Lys190, Ser220, Arg177, Trp176, Trp189, and Asp175, occupying the key binding site of CD38 with NAD^+^ and thwarting their binding (Figure 3c).

LPFLF forms two hydrogen bonds with the Thr221 residue of the CD38, with bond lengths of 3.0 Å and 3.1 Å, respectively. It also forms fourteen hydrophobic interactions with the residues Pro174, Ser224, Trp125, Trp176, Asn183, Thr158, Lys129, Asp219, Val185, Lys190, Asp156, Trp189, Ser186, and Leu157, occupying the key binding site of CD38 with NAD^+^ and obstructing their binding (Figure 3d).

VPPFY forms one hydrogen bond of 2.4 Å with the Ser224 residue of CD38, one hydrogen bond of 2.5 Å with the Asp219 residue, one hydrogen bond of 3.2 Å with the Thr221 residue, and one hydrogen bond of 2.1 Å with the Asn182 residue. It also forms eleven hydrophobic interactions with the residues Lys178, Lys190, Pro174, Val225, Ser181, Trp176, Ser220, Arg177, Trp189, Val185, and Asp175, occupying the key binding site of CD38 with NAD^+^ and preventing their binding (Figure 3e).

#### 2.5.2. The Docking of Polypeptides with Keap1

**Figure 4:**
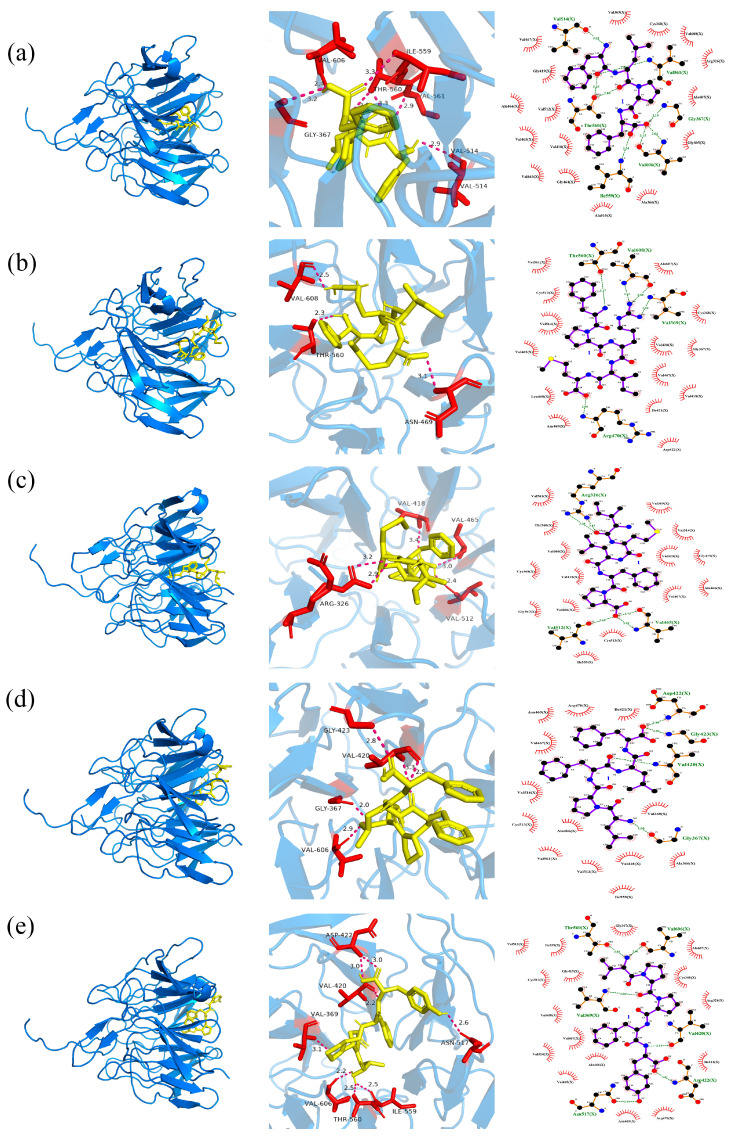
3D and 2D docking diagrams of FLPF (**a**), FPRIM (**b**), IMLFP (**c**), LPFLF (**d**), and VPPFY (**e**) with the active site of Keap1. The yellow part in the figure represents the ligand, the red part represents the residues of the receptor, and the purple dotted lines represent hydrogen bonds.

The Keap1–Nrf2 interaction exerts a substantial and crucial role in the modulation of oxidative stress. FLPF establishes a hydrogen bond with a length of 3.3 Å with the Ile559 residue of Keap1. Additionally, it forms a hydrogen bond of 3.2 Å with the Gly367 residue, a 2.4 Å hydrogen bond with the Val606 residue, a 2.9 Å hydrogen bond with the Val514 residue, a 2.9 Å hydrogen bond with the Val561 residue, and two hydrogen bonds of 3.3 and 2.8 Å with the Thr560 residue. Moreover, it engages in 16 hydrophobic interactions with the residues Val467, Val369, Cys368, Val608, Gly419, Arg326, Ala466, Ala607, Val512, Val465, Val418, Gly605, Val463, Gly464, Ala510, and Ala366 (Figure 4a).

FPRIM forms a 3.1 Å hydrogen bond with the Asn469 residue of Keap1, a 2.3 Å hydrogen bond with the Thr560 residue, and a 2.5 Å hydrogen bond with the Val608 residue. It also participates in 14 hydrophobic interactions with the residues Val561, Ala607, Cys513, Val514, Cys368, Val465, Val420, Gly367, Val467, Leu468, Ile421, Val418, Asn469, and Asp422 (Figure 4b).

IMLFP forms two hydrogen bonds with the Arg326 residue of Keap1 at distances of 2.9 Å and 3.2 Å, one hydrogen bond with the Val418 residue at a distance of 3.4 Å, one hydrogen bond with the Val465 residue at a distance of 3.0 Å, and one hydrogen bond with Val512 at a distance of 2.4 Å. It also forms 15 hydrophobic interactions with residues Val561, Val369, Thr560, Val514, Val608, Val420, Gly416, Cys368, Val418, Val467, Ala466, Gly367, Val606, Ile559, and Cys513 (Figure 4c).

LPFLF forms a 2.9 Å hydrogen bond with the Val606 residue of Keap1, a 2.0 Å hydrogen bond with the Gly367 residue, two hydrogen bonds of 3.3 and 2.5 Å with the Val420 residue, and a 2.8 Å hydrogen bond with the Gly423 residue. It also conducts 13 hydrophobic interactions with the residues Asn469, Arg470, Ile421, Val467, Val514, Val369, Cys513, Ala466, Val561, Val512, Val418, Asn366, and lle559 (Figure 4d).

VPPFY forms two hydrogen bonds with the Asp422 residue of Keap1 at distances of 3.0 Å each. It also forms one hydrogen bond with the Val420 residue at a distance of 2.2 Å, one with the Val369 residue at 3.1 Å, one with the Val606 residue at 2.2 Å, one with the Thr560 residue at 2.5 Å, one with the Ile559 residue at 2.5 Å, and one with the Asn517 residue at 2.6 Å. Additionally, VPPFY engages in 16 hydrophobic interactions with residues Val512, Ile559, Gly367, Ala607, Cys513, Gly419, Cys368, Val608, Arg326, Val467, Val514, Ala466, Ile421, Val465, Asn469, and Arg470 (Figure 4e).

### 2.6. Identification of the Antioxidant Activity of Peptides

**Figure 5:**
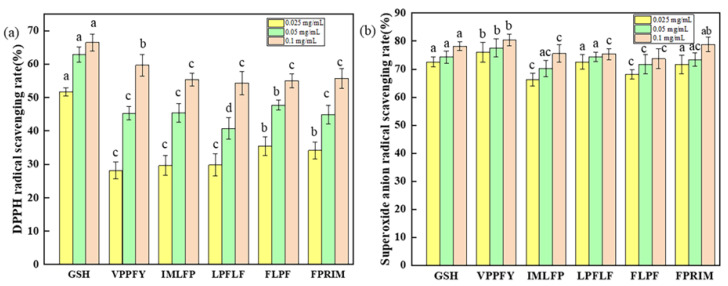
(**a**) DPPH radical scavenging activity of synthetic polypeptides; (**b**) superoxide anion scavenging activity of synthetic polypeptides. Different lowercases above the error bar suggest obvious differences in antioxidant activity of separated components (*p* < 0.05).

The tandem mass spectrometry (MS/MS) spectra of the five synthesized polypeptides are illustrated in Figure S2. Utilizing the commercial antioxidant GSH as a reference, it was observed that with the elevation in peptide concentration, the scavenging efficacy against DPPH free radicals was augmented. Among the peptides under investigation, VPPFY manifested the most prominent scavenging effect on free radicals, attaining a scavenging rate of approximately 60% with respect to DPPH at a concentration of 0.1 mg/mL (Figure 5a). These five peptides also demonstrated a relatively robust scavenging effect on superoxide anion radicals. Even at a relatively low concentration of 0.025 mg/mL, the scavenging rate of superoxide anion radicals exceeded 60%, and the effect was proximate to that of the commercial antioxidant GSH (Figure 5b).

## 3. Discussion

The study used ultrafiltration to fractionate the Y-1 hydrolysate into three fractions (>8 kDa, 5–8 kDa, and <5 kDa), with the <5 kDa fraction showing the optimal antioxidant activity (over 70% DPPH radical scavenging rate), outperforming both higher-molecular-weight fractions and 10 mg/mL Vc. This <5 kDa fraction was further separated via a Sephadex G-25 chromatographic column into six absorption peaks, whose eluates were collected, freeze-dried, and tested for activity: Peak 3 exhibited the most significant DPPH scavenging effect (surpassing 10 mg/mL Vc) and notable inhibitory activity against pancreatic cholesterol esterase (64.0%, exceeding 4 mg/mL sodium taurocholate) and pancreatic lipase (44.1%), while Peak 2 showed the highest α-glucosidase inhibitory activity (29.6%, comparable to 1 mg/mL acarbose).

Bin Wang isolated seven purified peptides with antioxidant activity from *Mytilus edulis*. At a concentration of 5.0 mg/mL, their DPPH radical scavenging rates could reach 50% to 85%. The DPPH radical scavenging rate of BNH-P7 (YPPAK) with the highest activity could reach 85.83%. It is composed of five amino acids, with a molecular weight of 574 Da, and belongs to short peptides. The proline it contains is closely related to antioxidant activity, which is similar to our results [26]. Qian Wang used proteases, pepsin, trypsin, alkaline protease, and papain to extract polypeptides from *Mytilus edulis*, which have strong in vitro scavenging abilities against free radicals such as OH, DPPH, and ABTS, and they showed a dose-dependent relationship. Although our results were less effective than those of the alkaline protease, they are comparable to the activities of other enzymes [17]. In a study investigating pigeon pea enzymatic protein hydrolysates and ultrafiltration peptide fractions as potential sources of antioxidant peptides, the DPPH radical scavenging EC_50_ values of sample hydrolysates were found to range from 0.91 to 2.44 mg/mL, with the trypsin-derived protein hydrolysate (PPHPa) exhibiting the highest activity (EC_50_ = 1.33 mg/mL); variations in DPPH scavenging capacity among different molecular weight fractions were ascribed to factors such as enzyme specificity, degree of hydrolysis, and peptide size [27]. These findings align with the results of the present study, supporting the conclusion that low-molecular-weight peptides (MW < 3 kDa) generally exhibit enhanced antioxidant potential.

Bioactive peptides can inhibit key metabolic enzymes, such as pancreatic lipase and cholesterol esterase involved in lipid–substrate hydrolysis. This provides a new and promising strategy for controlling hyperlipidemia and obesity. When compared to synthetic drugs like orlistat and simvastatin, bioactive peptides offer the advantages of lower cost and higher efficacy, thereby holding substantial potential in the domain of lipid metabolism and cholesterol reduction. Zhuangwei Zhang and colleagues determined the residue characteristics of pancreatic lipase and pancreatic cholesterol esterase through virtual screening and sequence analysis, thereby laying a theoretical groundwork for the development of new lipid-lowering peptide drugs [28]. Kewei Zheng and others enzymatically hydrolyzed defatted Antarctic krill and found that the enzymatic hydrolysis products of neutral protease and trypsin showed excellent inhibitory effects on α-glucosidase, with the inhibition rates being 34.28% and 33.6%, respectively. Subsequently, the selected 27 peptides were used for an in-depth study of their binding affinities with α-glucosidase. He believed that these inhibitory peptides mainly exerted their effects by binding to the active sites of α-glucosidase through hydrogen bonds, electrostatic interactions, and hydrophobic interactions [29].

Computers enable high-throughput evaluation of antioxidant peptide activity via bioinformatics databases, offering a cost-effective, rapid, and accurate approach for initial bioactive peptide screening. Molecular docking, a cornerstone of drug design, examines receptor–ligand interactions by evaluating spatial complementarity and energy matching, yielding critical parameters such as binding energy, intermolecular forces, and binding site details. Lower binding energy indicates stronger binding propensity, while intermolecular forces—visualizable through various computational tools—reveal interaction modalities [30]. Three primary docking modes are commonly used: rigid, semi-flexible, and flexible docking [31]. In recent years, molecular docking combined with molecular dynamics simulations has become indispensable for characterizing the molecular mechanisms of food-derived peptide bioactivity [32]. In this study, 384 peptide segments were identified in Peak 3 via LC-MS/MS, most of which were small peptides composed of five to six amino acids. Through computer simulations—incorporating molecular weight, hydrophobicity, and molecular docking binding energy with CD38—five candidate peptides with excellent activity were selected. Docking analyses with CD38 and Keap1 revealed that these peptides bind tightly via hydrogen bonds and hydrophobic interactions, participating in the regulation of oxidative stress. Furthermore, Ekambaram Gayathiri et al. [33] used docking to identify 10 diabetes-related genes (e.g., MTOR, CASP3) and their target proteins, providing multi-target candidates for diabetes research. Molecular docking has contributed significantly to diverse fields, including hypoglycemic, anti-inflammatory, antihypertensive, antioxidant, antibacterial, anti-aging, and anticancer research, underscoring its utility in marine drug discovery and development [33,34,35,36,37].

Natural foods serve as rich reservoirs of bioactive compounds, with peptides derived from animal, marine, plant, and insect sources garnering significant attention for their diverse biological activities, including antioxidant, anti-inflammatory, anticancer, antihypertensive, and antidiabetic properties. For instance, Junbin Guo et al. [25] isolated antioxidant peptides (GHVAA, MGKAA, VISGA, VLSGA) from Guangdong glutinous rice wine, which bind to CD38 to block NAD^+^ entry into its hydrophobic cavity, thereby inhibiting oxidative reactions in vivo. Chaonan Sun et al. [38] screened five polypeptides from fermented fish sauce, among which FS4-3 (MNPPAASIK) showed the highest DPPH scavenging activity (7.72%). Notably, the antioxidant capacity of the peptides in this study exceeds those reported sequences. All five synthesized peptides exhibit DPPH radical scavenging activity and superoxide anion radical scavenging activity. Among them, VPPFY achieves a scavenging rate of 60% and above at 0.1 mg/mL, which is close to the effect of the commercial antioxidant glutathione (GSH). These naturally derived bioactive peptides hold great potential in the development of new-generation health-promoting drugs, highlighting their translational value in the research and development of functional foods and pharmaceuticals.

## 4. Materials and Methods

### 4.1. Materials and Reagents

#### 4.1.1. Materials

The marine strain of *B. velezensis* Z-1 was isolated from the deep-sea sludge in the vicinity of Qingdao, Shandong Province. Mussels (*Mytilus edulis*) were procured from the local seafood market in Lianyungang. Subsequently, the mussel meat was carefully peeled, dried, and pulverized to obtain a dry powder, which was then stored at −20 °C in a refrigerator for future utilization.

#### 4.1.2. Reagents

1,1-Diphenyl-2-picrylhydrazyl (DPPH) was purchased from Beijing Wanjia Gene (Beijing, China). FeSO_4_·7H_2_O, H_2_O_2_ and salicylic acid were from Shanghai Aladdin (Shanghai, China). Pancreatic lipase, pancreatic cholesterol esterase, orlistat, acarbose, and α-glucosidase were from Sinopharm (Beijing, China). Tris, Vc, sodium taurocholate, and HCl were from Shanghai Sangon Biotech (Shanghai, China). Sephadex G-25 was bought from Shanghai Yuanye Biotech (Shanghai, China). The microplate reader was from Thermo Fisher Scientific (Waltham, MA, USA), the centrifuge from Eppendorf AG (Hamburg, Germany), and the freeze dryer from Ningbo Scientz (Ningbo, China). LC-MS/MS was conducted by Dangang Biotech (Wuhan, China). The capillary HPLC and Orbitrap mass spectrometer were both from Thermo Fisher Scientific (Waltham, MA, USA).

### 4.2. Preparation, Separation, and Purification of Mussel Bioactive Peptide

#### 4.2.1. Bioactive Peptide Preparation

Mussel bioactive peptides were prepared as described in previous research [19]. Specifically, dried mussel powder was mixed with ultrapure water at a 20:1 ratio (20 g powder in 400 mL water), followed by addition of 1 g *Bacillus velezensis* Z-1 protease. Enzymatic hydrolysis was conducted in a shaking incubator at 40 °C, 180 rpm for 40 h to yield hydrolysate Y-1.

#### 4.2.2. Ultrafiltration

The enzymatic hydrolysis product obtained was subjected to centrifugation, and the resultant supernatant was collected. Subsequently, ultrafiltration membranes with molecular weight cutoffs of 5 kDa and 8 kDa were employed for the fractionation process. Through this, three distinct components were ultimately acquired, namely those with molecular weights > 8 kDa, within the range of 5–8 kDa, and <5 kDa.

#### 4.2.3. Separation and Purification via Gel Filtration Chromatography

A Sephadex G-25 chromatographic column (with a column dimension of 100 cm × 1.6 cm) was employed for the separation of the fraction exhibiting the highest DPPH scavenging activity. When the sample volume was adjusted to 10 mL (at a concentration of 50 mg/mL), the pump flow rate was precisely controlled and set at 1 mL/min, and elution was performed using distilled water. The absorbance values of the collected solutions were measured at 280 nm. Subsequently, based on the obtained measurement results, the absorption peaks were plotted, and the fractions corresponding to each absorption peak were meticulously collected.

### 4.3. Determination of Antioxidant Activity

#### 4.3.1. Determination of DPPH Scavenging Activity

The assay for determining DPPH scavenging activity was conducted by referring to the methodology proposed by previous research [39]. Three ultrafiltration fractions were used in the DPPH scavenging experiment. Procedure: Mix 1 mL mussel bioactive peptide solution with 1 mL 0.1 mM DPPH ethanolic solution, and incubate in the dark for 30 min. Blank group: Replace the peptide with ultrapure water and DPPH with pure ethanol. Control group: Replace the sample with ultrapure water. Take V_c_ as the positive control. Measure each group’s absorbance at 517 nm and calculate sample’s DPPH scavenging activity using the following formula:

DPPH scavenging rate (%) = [1 − (A_b_ − A_c_)/(A_a_ − A_c_)] × 100% (1)

where A_a_ represents the absorbance of the control group (ultrapure water + DPPH ethanol solution system), A_b_ represents the absorbance of the experimental group (mussel bioactive peptide + DPPH ethanol solution system), and A_c_ represents the absorbance of the blank group (ultrapure water + pure ethanol system).

#### 4.3.2. Determination of Superoxide Anion Scavenging Activity

The experimental procedure was carried out by referring to the methodology proposed by previous research [39]. A total of 0.2 mL of various concentrations of polypeptide aqueous solutions were mixed with 1 mL of 50 mM Tris-HCl and incubated at 25 °C for 10 min. Then, 30 μL of 6 mM pyrogallol was added and let stand at room temperature for 30 min, and absorbance was measured at 325 nm. Control: The polypeptide was replaced with pure water. Blank: Pyrogallol was replaced with HCl. The effectiveness of the experimental system was verified using the commercial antioxidant GSH. The calculation formula was as follows:

Superoxide anion radical scavenging rate (%) = [1 − (A_a_ − A_b_)/A_c_] × 100% (2)

where A_a_ represents the absorbance of the sample group (including the reaction system with peptides and pyrogallol), A_b_ represents the absorbance of the blank group (the background absorbance of the system containing peptides but without pyrogallol), and A_c_ represents the absorbance of the control group (the absorbance of the system without peptides but with pyrogallol).

### 4.4. Determination of Lipid-Lowering Ability

#### 4.4.1. Determination of Pancreatic Cholesterol Esterase Inhibitory Activity

The experimental protocol was implemented with reference to the methodology proposed by Fangfang Huang, accompanied by minor modifications [40]. The sample was diluted to 1 mg/mL. A total of 0.1 M pH 7.2 PBS, 5 mM/mL pNPB, And pancreatic cholesterol esterase at 5 μg/mL were prepared. Blank: PBS for enzyme. Control: No sample in PBS. Blank control: PBS with enzyme, no sample. All were incubated at 37 °C for 30 min, and absorbance was measured at 405 nm. Sodium taurocholate (STC) was taken as the positive control. The pancreatic cholesterol esterase inhibitory activity was computed using the following formula:

Pancreatic cholesterol esterase inhibitory activity (%) = [1 − (A_c_ − A_d_)/(A_a_ − A_b_)] × 100% (3)

where A_a_ represents the absorbance of the control group (PBS + enzyme solution + pNPB), A_b_ represents the absorbance of the blank control group (PBS + pNPB), A_c_ represents the absorbance of the sample group (PBS + enzyme solution + pNPB + sample), and A_d_ represents the absorbance of the sample blank group (PBS + sample).

#### 4.4.2. Determination of Pancreatic Lipase Inhibitory Activity

The experimental procedure was carried out in accordance with the approach of Fangfang Huang, with slight alterations [40]. The sample was diluted to 1 mg/mL. A total of 0.1 M pH 7.2 PBS, 5 mM/mL pNPB, and pancreatic lipase at 5 μg/mL were prepared. Blank: PBS for lipase. Control: No sample in PBS. Blank control: No sample or lipase in PBS. All were incubated at 37 °C for 30 min. Absorbance was measured at 405 nm. Orlistat was taken as the positive control. The pancreatic lipase inhibitory activity was calculated using the following formula:

Pancreatic lipase inhibitory activity (%) = [1 − (A_c_ − A_d_)/(A_a_ − A_b_)] × 100% (4)

where A_a_ represents the absorbance of the control group (PBS + enzyme solution + pNPB), A_b_ represents the absorbance of the blank control group (PBS + pNPB), A_c_ represents the absorbance of the sample group (PBS + enzyme solution + pNPB + sample), and A_d_ represents the absorbance of the sample blank group (PBS + sample).

### 4.5. Determination of α-Glucosidase Inhibitory Activity

The experimental method was conducted with reference to Jingfei Hu’s approach, with minor modifications [41]. The sample was diluted to 1 mg/mL. A total of 2.5 mM/mL pNPG and 5 μg/mL α-glucosidase were prepared. Control: Ultrapure water was used instead of the sample. Blank: No pNPG or α-glucosidase. All were incubated for 30 min. Absorbance was measured at 405 nm. Acarbose was taken as the positive control. The α-glucosidase inhibitory activity of the sample was calculated using the following formula:

α-Glucosidase inhibitory activity (%) = [1 − (A_b_ − A_c_)/A_a_] × 100% (5)

where A_a_ represents the absorbance of the control group (PBS + α-glucosidase + pNPG system), A_b_ represents the absorbance of the experimental group (PBS + α-glucosidase + pNPG + sample system), and A_c_ represents the absorbance of the blank group (PBS + sample system).

### 4.6. Identification of Peptide Fractions

The sample from the peak of highest comprehensive activity was sent to WuhanDangang for sequencing. It then underwent LC-MS/MS analysis. Column: 150 μm × 150 mm, 1.9 μm 100 Å Acclaim PepMap RPLC C18. Mobile phase A: 0.1% FA. B: 0.1% FA + 80% ACN. Flow: 600 nL/min. Each fraction was analyzed in 66 min. Primary MS: Res 70,000, AGC 3e6, max IT 100 ms, scan 300–1800 m/z. Secondary MS: Res 17,500, AGC 1e5, max IT 50 ms, top N 20, NCE/step 28. Byonic searched original MS files vs. target DB. Params: Fixed C, Var M, Enz non-spec, Miss 3, PMT 20 ppm, FMT 0.02 Da.

### 4.7. Screening of Bioactive Peptides

Peptides were ranked by descending comprehensive scores from PeptideRanker, prioritizing antioxidant activity. PepDraw (http://www.pepdraw.com/, accessed on 17 March 2024) was used to analyze amino acid composition, molecular size, and hydrophobicity for initial screening.ToxinPred (https://webs.iiitd.edu.in/raghava/toxinpred/, accessed on 17 March 2024) was used to evaluate peptide toxicity for the elimination of toxic peptides. Molecular docking of pre-screened peptides with CD38 and Keap1 was performed using AutoDock software. CD38 (PDB: 3DZK) and Keap1–Kelch (PDB: 2FLU) receptors were obtained from the Protein Data Bank. Peptide charges were calculated via AutoDockTools 1.5.6, and PDBQT files were docked using Vina. Docking results were visualized with Ligplot and Pymol to analyze interaction forces. The top five peptides with the highest binding energy and key amino acid binding sites were synthesized by Shanghai Shenggong.

### 4.8. Peptide Synthesis

The selected peptides were synthesized using a solid-phase synthesis procedure and purified by reverse-phase high-performance liquid chromatography (RP-HPLC).

### 4.9. Verification of the Antioxidant Activity of Polypeptides

The five peptides possessing potential antioxidant activity were individually diluted to concentrations of 0.025 mg/mL, 0.05 mg/mL, and 0.1 mg/mL for the purpose of assessing their capabilities in scavenging DPPH free radicals and superoxide anion radicals. Subsequently, a comparison was made with commercially available glutathione (GSH), which is known for its antioxidant properties, in order to authenticate their antioxidant activities.

### 4.10. Data Analysis Method

All experiments were replicated three times. The data were expressed as mean ± standard deviation (±SD). Statistical analysis was conducted using SPSS 17.0 statistical software. A *p*-value of less than 0.05 was regarded as the threshold for statistical significance.

## 5. Conclusions

In the present study, the protein hydrolysate Y-1 was successfully acquired through the hydrolysis of mussels mediated by *B. velezensis* Z-1. Subsequently, five polypeptides possessing antioxidant activity, namely FLPF, IMLFP, LPFLF, FPRIM, and VPPFY, were meticulously screened out. Notably, VPPFY demonstrated the most remarkable scavenging efficacy against free radicals, attaining a DPPH scavenging rate of approximately 60% at a concentration of 0.1 mg/mL. These five peptides also manifested a relatively potent scavenging effect on superoxide anion radicals. Even at a relatively low concentration of 0.025 mg/mL, the scavenging rate of superoxide anion radicals exceeded 60%, approaching the effect of the commercial antioxidant GSH. This study offers valuable insights and guidance for the application of *B. velezensis* Z-1 protease in food processing, the development of high-value mussel meat, and the exploration of mussel peptides with potential antioxidant activity. In the future, mussel peptides could be incorporated as natural antioxidants into health foods or beverages to delay oxidative deterioration [42]. Furthermore, they might also function as auxiliary components in drugs for treating chronic diseases, which is expected to become a promising strategy for alleviating organ damage caused by oxidative stress [43]. It should be emphasized that this study has only confirmed the antioxidant activity of the target peptides and their inhibitory effects on related enzymes through in vitro experiments and has not yet verified their targeted distribution in vivo. Given that there may be significant differences between in vitro activity and actual in vivo efficacy, this study has certain limitations. Therefore, the specific health-promoting effects of these peptides still need to be further verified in subsequent studies through systematic animal experiments (such as detecting antioxidant indicators in the body and analyzing metabolism-related parameters).

## Figures and Tables

**Table 1:** Screening of antioxidant peptides.

Name	A	Length	B	C	D	E	F
FLPF	523.3	4	49.5	100	3.37	−8.3	−9.7
IMLFP	620.3	5	52.9	100	3.29	−7.6	−7.9
LPFLF	636.4	5	59.9	100	2.12	−9.1	−8.3
FPRIM	663.4	5	35.7	80	6.35	−7.8	−7.7
VPPFY	622.3	5	37.5	80	5.30	−9.1	−9.5

Explanation: Within the table, A stands for observed *m*/*z*, which is the mass-charge ratio actually detected by the peptide; B designates the separation time (s); C denotes the percentage of hydrophobic amino acids (%); D represents hydrophobicity (kcal/mol); E represents the binding energy in the docking process with CD38 (kcal/mol); and F stands for the binding energy in the docking with Keap1 (kcal/mol).

## Data Availability

The data presented in this study are available in the present article.

## Supplementary Materials

The following supporting information can be downloaded at: https://www.mdpi.com/article/10.3390/md23080294/s1, Figure S1: Chromatogram of protein with absorption peak 3; Figure S2: The secondary mass spectra of synthetic polypeptides FLPF (a), FPRIM (b), IMLFP (c), LPFLF (d), and VPPFY (e); Table S1: Partial peptide sequences identified by LC-MS/MS.
