# Supplementary material for: Isolation and Purification of Novel Antioxidant Peptides from Mussel (Mytilus edulis) Prepared by Marine Bacillus velezensis Z-1 Protease
Source: Mar Drugs. 2025 Jul 23;23(8):294. doi: 10.3390/md23080294 (PMC12387240; doi:10.3390/md23080294)
Supplement: Supplementary file 1 [file marinedrugs-23-00294-s001.zip › marinedrugs-3751022-supplementary.pdf]

Supplementary materials

Figure S1

BTP20220610-10-02：

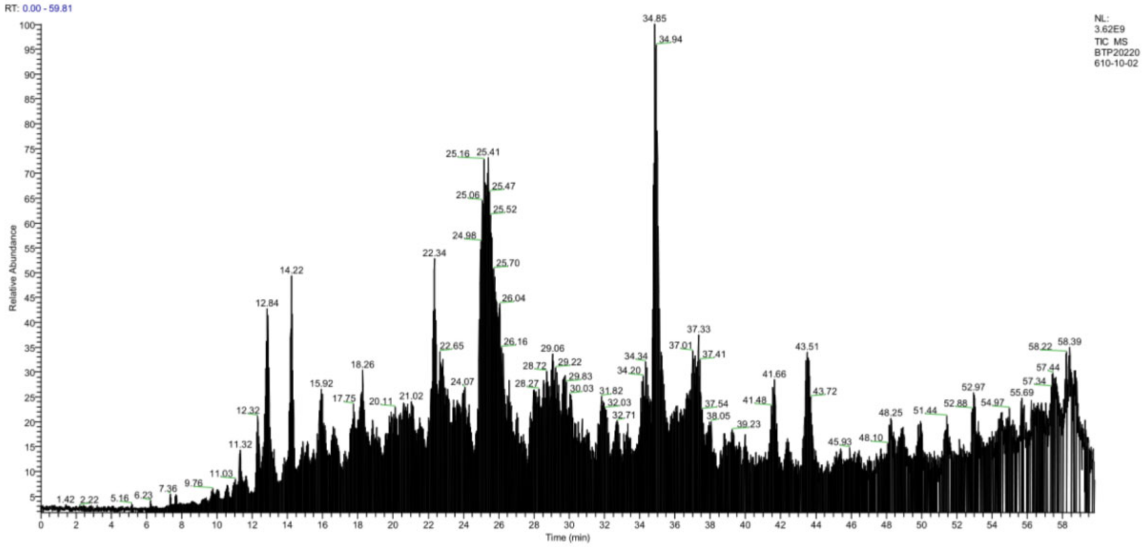

Figure S1 Chromatogram of protein with absorption peak 3.

Figure S2

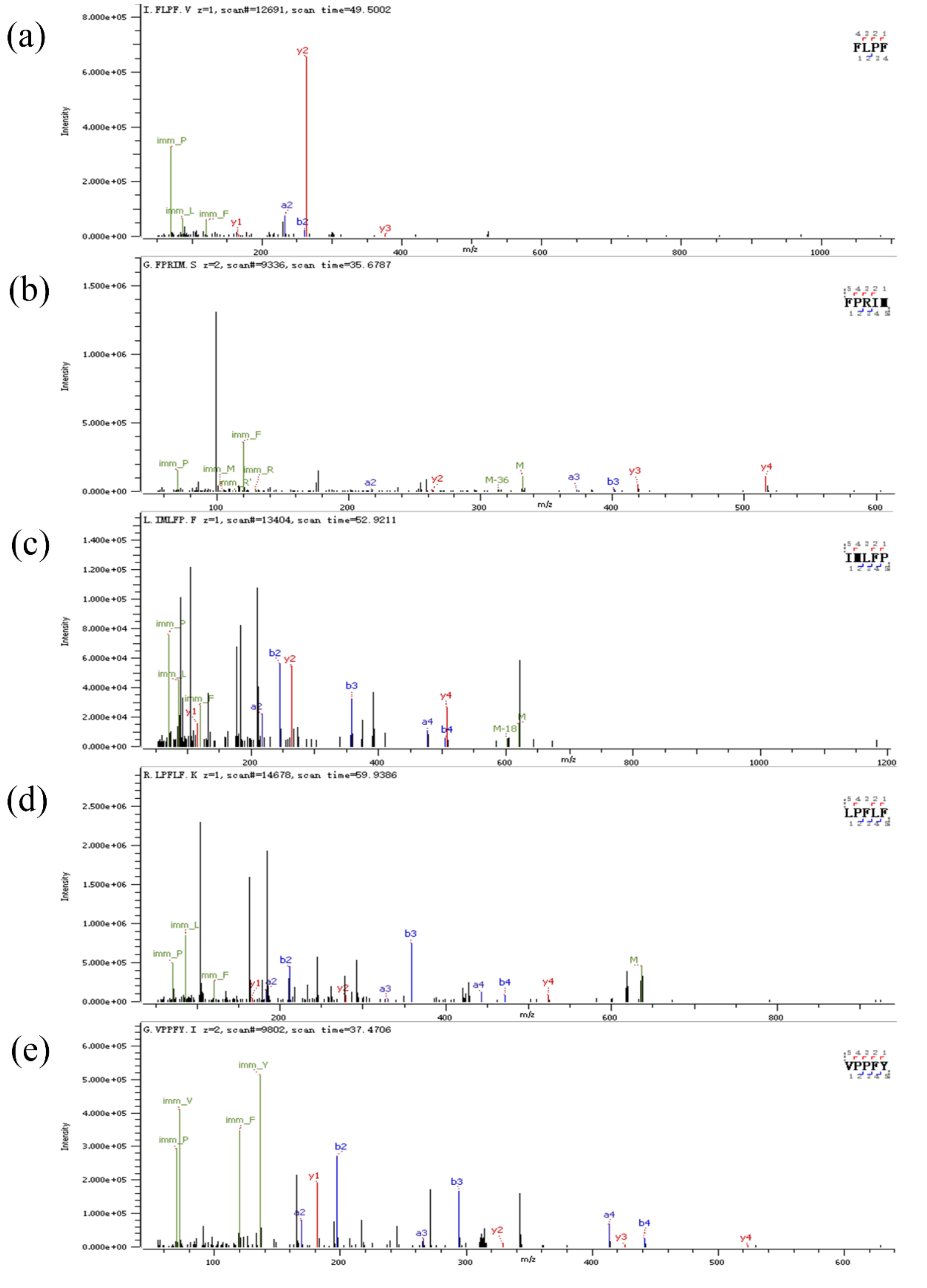

Figure S2. The secondary mass spectra of synthetic polypeptides FLPF(a), FPRIM(b), IMLFP(c), LPFLF(d), and VPPFY(e)

Table S1

Table S1 Partial peptide sequences identified by LC-MS/MS

Name	m/z value	Score	Retention time (s)	Amino acid abundance
TERGYSF	430.202	475.9	23.243	48537000
RGPFH	307.164	365.3	9.5353	11691000
DLRFQ	339.682	343.8	23.6806	9041300
VPPFY	311.665	336.8	37.4706	17445000
SPRPY	310.163	329.2	11.0649	2968900
SYPYHS	377.169	296.0	10.1096	17334000
HYNDPFYGK	570.762	296.0	23.8012	30269000
TWNDPR	394.689	295.2	15.5449	41088000
YNDPFYGK	502.232	294.1	27.8544	16043000
LAGY	423.225	285.5	20.8251	14202000
TYRQL	340.690	285.0	14.6488	9056800
TPRWK	344.195	276.7	19.3679	37496000
PGFP	417.214	269.6	23.3069	11790000
VGPF	419.230	268.6	29.4832	50535000
GPFHF	302.647	268.1	35.0168	18109000

Elucidation: The m/z value as observed within the table pertains to the experimen tally detected mass-to-charge ratio of the peptide. The computed mass (M+H) presente d in the table corresponds to the theoretical molecular weight of the peptide moiety. I n the tabular data, the mass error, quantified in parts per million (ppm), reflects the d eviation in molecular weight. The score denoted in the table represents the quantitative assessment or rating of the peptide. The intensity value within the table is indicative of the relative abundance or prevalence of the peptide. The scan time specified in the table corresponds to the retention time of the peptide during the analytical process.
